# Insertion site of central venous catheter among hospitalized adult patients: A systematic review and network meta-analysis

**DOI:** 10.3389/fmed.2022.960135

**Published:** 2022-08-29

**Authors:** Masaaki Sakuraya, Hiromu Okano, Shodai Yoshihiro, Shoko Niida, Keina Kimura

**Affiliations:** ^1^Department of Emergency and Intensive Care Medicine, JA Hiroshima General Hospital, Hatsukaichi, Japan; ^2^Department of Critical and Emergency Medicine, National Hospital Organization Yokohama Medical, Yokohama, Japan; ^3^Department of Pharmacy, Onomichi General Hospital, Onomichi, Japan

**Keywords:** central venous catheter, complication, hospitalization, insertion site, network meta-analysis

## Abstract

**Introduction:**

Central venous catheterization is a commonly performed procedure, accounting for approximately 8% of hospitalized patients. Based on the current literatures, the most acceptable site for central venous catheterization is inconclusive, considering various complications in hospitalized patients. Herein, we conducted a network meta-analysis to assess the clinically important complications among internal jugular, subclavian, femoral, and peripheral insertion.

**Materials and methods:**

The Cochrane Central Register of Controlled Trials, MEDLINE, Web of Science, Ichushi databases, Clinicaltrials.gov, and International Clinical Trials Registry Platform were searched. Studies including adults aged ≥ 18 years and randomized control trials that compared two different insertion sites (internal jugular, subclavian, femoral, and peripheral vein) were selected. The primary outcomes were clinically important infectious, thrombotic, and mechanical complications.

**Results:**

Among the 5,819 records initially identified, 13 trials (6,201 patients) were included for a network meta-analysis. For clinically important infectious complication, subclavian insertion decreased the complication risk, compared with internal jugular [risk ratio (RR), 0.30; 95% confidence interval (CI), 0.11–0.81; moderate certainty], and femoral insertion increased than subclavian insertion (RR 2.56; 95% CI, 1.02–6.44; moderate certainty). Peripheral insertion was also significantly associated with a lower risk compared with internal jugular (RR 0.06; 95% CI, 0.01–0.32; low certainty); subclavian (RR 0.21; 95% CI, 0.05–0.77; moderate certainty); and femoral insertion (RR 0.08; 95% CI, 0.02–0.40; low certainty). For clinically important thrombotic complication, we did not find significant differences between insertion sites. For clinically important mechanical complication, femoral insertion decreased the complication risk, compared with internal jugular (RR 0.42; 95% CI, 0.21–0.82; moderate certainty) and subclavian insertion (RR 0.33; 95% CI, 0.16–0.66; moderate certainty). Peripheral insertion was also associated with the lower complication risk compared with internal jugular (RR 0.39; 95% CI, 0.18–0.85; low certainty) and subclavian insertion (RR 0.31; 95% CI, 0.13–0.75; moderate certainty).

**Conclusion:**

The insertion site of the central venous catheter, which is most likely to cause the fewest complications, should be selected. Our findings can provide the rationale for deciding the insertion site for a central venous catheter.

**Systematic review registration:**

[www.protocols.io], identifier [61375].

## Introduction

Secure and reliable venous catheterization is the cornerstone of managing hospitalized patients. Generally, peripheral catheters are preferred, as they are generally safer, easier to insert, and less painful than central catheters. Centrally inserted central venous catheters (CICCs) are often placed in patients who are administered key intravenous drugs, including vasoactive drugs and chemotherapy. Furthermore, patients without arteriovenous fistulas who receive renal replacement therapy also require central venous access. Central venous catheterization is a commonly performed procedure, accounting for approximately 8% of hospitalized patients (1), and more than five million CICCs are inserted in the United States each year (1, 2). The anatomic site chosen for CICC placement, including the jugular, subclavian, and femoral veins, influences the risk and type of complications (3). Recently, peripherally inserted central venous catheters (PICC) have been used as substitutes for CICC in an increasing number of hospitalized patients (4).

The central venous catheter insertion site that is most likely to cause the fewest complications should be selected, considering complication risks in individual cases, since baseline risks also depend on the operator experience, the expected duration of catheter placement, and patient risk factors (e.g., mechanical ventilation, hemostasis disorders, and obesity). Previous systematic reviews and meta-analyses have demonstrated that femoral insertion is not preferable for central venous access considering infectious and thrombotic complications (5, 6). Furthermore, a recent systematic review and network meta-analysis (NMA) revealed that subclavian insertion was associated with a lower colonization risk, but may be comparable to internal jugular insertion in terms of reducing catheter-related bloodstream infection risk (7). In addition to these complications, mechanical complications, including pneumothorax, hemothorax, arterial puncture, and hematoma also play an important role in determining the insertion site. However, very few meta-analyses have evaluated these three major complications among the insertion sites of central venous catheterization, including PICC.

Based on the current literature, the most acceptable site for central venous catheterization is inconclusive, considering various complications in hospitalized patients. Herein, we performed an NMA to evaluate three major complications related to central venous catheter in hospitalized patients.

## Materials and methods

### Protocol and registration

This systematic review was designed according to the Preferred Reporting Items for Systematic Review and Meta-Analyses extension statement for reviews incorporating NMAs (8), and the protocol was registered at protocols.io (Protocol integer ID 61375).

### Eligibility criteria

#### Type of studies

We included all reported randomized control trials (RCTs), regardless of the language and publication status (published, unpublished, and academic abstracts). Randomized crossover, cluster-randomized, or quasi-experimental trials were excluded.

#### Type of participants

This review included adults (aged ≥ 18 years) with short-term, non-cuffed, and non-tunneled central venous catheters inserted using maximal sterile barrier precautions. We included catheters used for monitoring, administering drugs, and dialysis, but not cannulas for extracorporeal membrane oxygenation. The current meta-analysis excluded studies with catheter exchange over the guidewire and the following devices: coated catheters (e.g., antimicrobial-impregnated, chlorhexidine/silver sulfadiazine, heparin), tunneled catheters, cuffed catheters, and venous access ports.

#### Types of interventions and comparators

We included RCTs that compared two of the following four insertion sites [(1) Internal jugular insertion, (2) Subclavian insertion, (3) Femoral insertion, (4) Peripheral insertion]. Any insertion technique, antiseptics, or any number of lumens was acceptable. When comparing CICC with PICC, we adopted the most indwelling insertion site.

#### Types of outcomes

The outcome measures included clinically important catheter-related infections, thrombotic complications, and mechanical complications during the observation period of each study. We defined a clinically important infectious complication as one with systemic symptoms other than local infection and no other obvious focus of infection (e.g., blood stream infection, sepsis), a clinically important thrombotic complication as one with clinical symptoms or that which requires treatment, and a clinically important mechanical complication as the one that requires procedures or careful observation (e.g., pneumothorax, hemothorax, hematoma, and bleeding).

### Information sources

We searched the following six databases for eligible trials: The Cochrane Central Register of Controlled Trials; MEDLINE *via* PubMed; Web of Science; Ichushi, a database of Japanese research papers; Clinicaltrials.gov; and World Health Organization International Clinical Trials Registry Platform.

### Search

We used the terms “critical illness,” “hospitals,” or “hospital units” AND “central venous catheterization,” “renal dialysis,” or “renal replacement therapy” AND “internal jugular,” “subclavian,” “femoral,” “peripherally inserted,” or “insertion site” in searches performed in March 2022 (details shown in Supplementary Table 1). We also included a filter in the search strategy to identify RCTs in PubMed, which is a sensitivity and precision-maximizing version.

### Study selection

We used machine learning algorithms for systematic reviews (high-sensitivity strategies) to identify RCTs^1^ (9). Two of the four physicians (HO, SY, SN, and KK) screened the title and abstract or the full text of the relevant studies, during the first and second screenings, respectively, and independently extracted data from the included studies into standardized data forms. Disagreements, if any, were resolved by discussion with one of four physicians who did not screen that particular study; the original authors were contacted for clarification as required. For abstract-only studies that could not be evaluated for eligibility based on our review criteria, we contacted the authors. Discrepancies between the two reviewers were resolved by mutual discussion or discussion with a third reviewer, as needed.

### Data collection process

After identifying the studies in the second screening, data was extracted from each study by the reviewers (HO, SY, SN, and KK) using two tools: the Cochrane Data Collection Form (RCTs only) (10) and Review Manager Software (RevMan version 5.4.1, The Cochrane Collaboration, 2014) (11). We contacted the authors with unknown data.

### Data items

We extracted the following study characteristics:

(1) Methods: The study design, total study duration, number, and locations of study centers, study setting, withdrawals, date of study initiation, and funding sources were reviewed.
(2) Participants: Number, age, sex, body mass index, setting, and inclusion/exclusion criteria.
(3) Interventions: Insertion site, catheter, duration of placement, operator experience, antiseptic, dressing, and insertion technique.
(4) Outcomes: Outcomes that were specific were collected and the timepoints reported.

### Geometry of the network

Network plots were constructed to determine the number of trials and patients included in this meta-analysis. We demonstrated a network geometry that presented the nodes as interventions and each head-to-head direct comparison as lines connecting these nodes. The size of the nodes was proportional to the number of participants in each node. The thickness of the connecting line was proportional to the number of randomized clinical trials for each comparison.

### Risk of bias within individual studies

The risk of outcome bias in the included studies was independently assessed by two of the five authors (MS, HO, SY, SN, and KK) using a modified version of the Cochrane “Risk of Bias” instrument (12). They assessed the overall risk of bias as the worst in any of the following domains: randomization process, deviations from intended interventions, missing outcome data, measurement of the outcomes, and selection of the reported results. The risk of bias was graded as “low risk of bias,” “some concerns,” or “high risk of bias.” Blinding was not achievable in trials comparing CICC insertion sites. Thus, we evaluated overall bias except for bias in the measurement of the outcome, which contributed to the judgment that overall bias was high risk in most trials. Discrepancies between the two reviewers were resolved through discussion among themselves or with a third reviewer as necessary.

### Planned methods of analyses

#### Direct comparison meta-analysis

A pairwise meta-analysis was performed using RevMan 5.3 (RevMan 2014) (11). Forest plots were used for meta-analysis, and the effect sizes were expressed as risk ratios (RRs) with 95% confidence intervals (CIs) for the categorical data. The outcome measures were pooled using a random-effects model to measure study-specific effects. For all the analyses, a two-sided *P* value < 0.05 was considered statistically significant.

#### Network comparison meta-analysis

Network meta-analysis was performed using a frequentist approach with multivariate random-effects meta-analysis using the mvmeta command in Stata 15.1 (StataCorp LLC, College Station, TX, United States). The network meta-command allowed us to fit consistency models and estimate network RRs for each treatment strategy based on both direct and indirect comparisons (13). Forest plots of the RRs with 95% CIs were constructed for each treatment strategy in the network.

Ranking plots (rankograms) were constructed based on the probability that a given treatment had the highest event rate for each outcome. The surface under the cumulative ranking curve (SUCRA), which is a simple transformation of the mean rank, was used to determine the treatment hierarchy (14). Higher SUCRA values, which range from 0 to 100%, increase the likelihood that a therapy is ranked amongst the best in an NMA (15).

### Assessment of inconsistency

Study heterogeneity among trials for each outcome was assessed by visually inspecting the forest plots and using the *I*^2^ statistic to quantify any inconsistencies (16). Publication bias was assessed visually using a funnel plot (15).

Coherence in NMA refers to the consistency in the estimates of treatment effects between direct and indirect comparisons (17). For each pairwise comparison, coherence was assessed using a node-splitting method (18). We also examined coherence globally across the network using the Wald chi-square test obtained by fitting the inconsistency model (13).

### Grades of recommendation, assessment, development, and evaluation working group assessments of the certainty of evidence for each network comparison

To assess the certainty of the evidence for direct comparisons, we used the standard GRADE methodology (19–21). At first, we evaluated the risk of bias, indirectness, inconsistency, and publication bias. However, we did not rate down for imprecision which was evaluated at a later step (22, 23). For indirect comparisons, we started with the lowest certainty of evidence for the contributing direct comparisons, and then rated it down if there was substantial intransitivity. The transitivity assumption underlying NMA was evaluated by comparing the distribution of clinical and methodological variables that could act as effect modifiers across treatment comparisons. We assessed the certainty in each network comparison considering the highest certainty of evidence between the direct and indirect evidence (23); the network estimate was subsequently rated considering the imprecision and incoherence (24, 25).

### Additional analyses

Pre-planned sensitivity analyses, which excluded trials comparing CICC in multiple sites with PICC, and which limited trials among critically ill patients, were performed to assess the robustness of the findings. In addition, we performed *post hoc* sensitivity analyses investigating the occurrence of infectious and thrombotic complications according to catheter indwelling duration. When significant incoherence was present in the outcomes, we also performed *post hoc* sensitivity analyses to explore the source.

## Results

### Study selection

**FIGURE 1:**
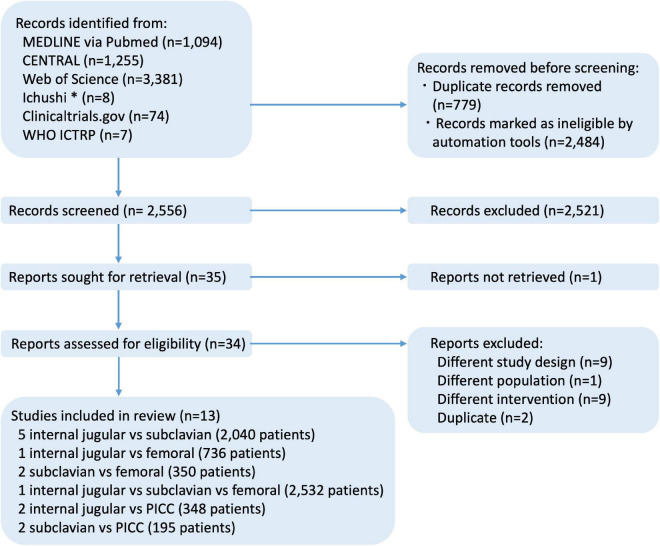
Flow diagram of studies included in this review. *Ichushi is a database of Japanese research papers. CENTRAL, cochrane central register of controlled trials; ICTRP, international clinical trials registry platform; MEDLINE, medical literature analysis and retrieval system on line; WHO, world health organization.

The search strategy identified 5,819 records, including 13 RCTs (6,201 participants; range: 61–2,532 participants) that were eligible for inclusion (Figure 1).

### Presentation of network structure and summary of network geometry

**FIGURE 2:**
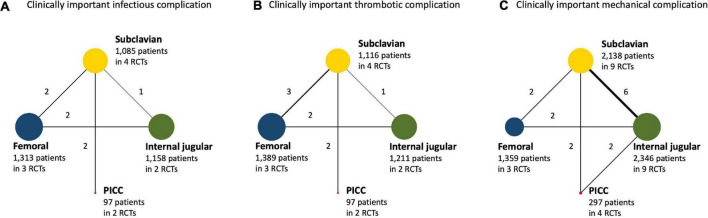
**(A)** Clinically important infectious complication. **(B)** Clinically important thrombotic complication. **(C)** Clinically important mechanical complication. Network plot for central venous access sites for hospitalized patients. When randomized control trials (RCTs) for direct comparisons exist, this is shown by connections between nodes. The size of the node represents the number of participants who received the intervention. The thickness of lines connecting nodes represents the number of trials for that comparison. PICC, peripherally inserted central venous catheter; RCT, randomized controlled trial.

Of the included trials that evaluated four different interventions, these included four of six potential head-to-head comparisons for clinically important infectious complications and thrombotic complications and five of six potential head-to-head comparisons for clinically important mechanical complications (Figure 2). Specifically, five trials compared subclavian with internal jugular insertion (26–30), one trial compared femoral with internal jugular insertion (31), two trials compared femoral with subclavian insertion (32, 33), two trials compared PICC with internal jugular insertion (34, 35), and two trials compared PICC with subclavian insertion (36, 37). In addition, a three-group study directly compared femoral, internal jugular, and subclavian insertions (3). However, no trials have compared PICC with femoral insertion. There were 15 comparisons among the 13 RCTs.

### Study characteristics and risk of bias assessment

Table 1 and Supplementary Table 2 show the participants, interventions, comparisons, outcomes, and cohort characteristics of the included trials. Most trials included critically ill or surgical patients, one of which investigated catheters for renal replacement therapy (31). Four trials comparing PICC with CICC included patients who required parenteral nutrition therapy or chemotherapy (34–37). The mean age at randomization ranged from 41 to 65 years, mean catheter placement days were 2.0–27.3 days for CICC and 9.6–115.1 days for PICC, experienced physicians and medical staffs performed the procedures in nine trials (3, 27, 28, 30–32, 34, 36, 37), ultrasound guidance was encouraged in five trials (3, 26, 30, 31, 37), and landmark technique was used in six trials (27–29, 34). Povidone-iodine and chlorhexidine were commonly used as antiseptics, and each insertion site was covered with a sterile transparent dressing in most trials. Most trials did not report the use of antibiotics or anticoagulants.

**TABLE 1:** Summary of characteristics of the studies included in the network meta-analysis.

Source	Funding	Total No. of catheter	Setting, population	Main exposure	Comparator	Outcomes of interest assessed	Time point
Cowl (36)	Not stated	102	Hospitalized patients who required TPN	PICC (*N* = 51)	Subclavian (*N* = 51)	CRBSI, clinically evident thrombophlebitis, mechanical complication (pneumothorax)	Until removal
Durbec (33)	Not stated	61	Critically ill patients with comatose or sedation	Femoral (*N* = 30)	Superior vena cava: subclavian (*N* = 21) or internal jugular (*N* = 10)	Venous thromboembolism	Until removal
Fournil (26)	Not stated	201	Critically ill patients	Subclavian (*N* = 101)	Internal jugular (*N* = 100)	Mechanical complication (hematoma, pneumothorax)	During insertion procedure
Gülmen (27)	None	94	Cardiac surgery	Subclavian (*N* = 45)	Internal jugular (*N* = 49)	Mechanical complication (hematoma, pneumothorax)	During insertion procedure
Guo (34)	Not stated	98	Colorectal cancer patients at a nutrition risk	PICC (*N* = 49)	Internal jugular (*N* = 49)	Mechanical complication (pneumothorax)	Not stated
Kocum (28)	Local university research funds	195	Cardiac surgery	Subclavian (*N* = 130)	Internal jugular (*N* = 65)	Mechanical complication (hematoma, pneumothorax, hemothorax)	Not stated
Laiq (29)	None	200	Cardiac surgery	Subclavian (*N* = 100)	Internal jugular (*N* = 100)	Mechanical complication (pneumothorax, hemothorax)	Not stated
Merrer (32)	Plastime laboratories and Smith and Nephew	289	Critically ill patients	Femoral (*N* = 145)	Subclavian (*N* = 144)	Major catheter related infectious complications, major catheter related thrombosis, major mechanical complication	4 days within catheter removal
Parienti (31)	Center Hospitalier Universitaire de Caen	736	Critically ill patients, RRT	Femoral (*N* = 370)	Internal jugular (*N* = 366)	CRBSI, symptomatic DVT, hematoma	4 days within catheter removal
Parienti (3)	French Ministry of Health and an unrestricted academic grant from the French Health Ministry	2,532	Critically ill patients	Femoral (*N* = 844), subclavian (*N* = 843)	Internal jugular (*N* = 845)	CRBSI, symptomatic DVT, mechanical complication (grade ≥ 3)*	48 h after catheter removal
Picardi (37)	Not stated	93	Acute myeloid leukemia	PICC (*N* = 46)	CICC: Subclavian (*N* = 35) or internal jugular (*N* = 12)	CRBSI, symptomatic thrombotic complication, mechanical complication (serious bleeding, pneumothorax)	30 days from insertion
Shin (30)	None	1,350	Surgical patients	Subclavian (*N* = 677)	Internal jugular (*N* = 673)	Mechanical complication (pneumothorax, hemothorax)	During insertion procedure
Zhong (35)	Not stated	250	Tumor patients	PICC (*N* = 151)	Internal jugular (*N* = 99)	Mechanical complication (hematoma, pneumothorax, hemothorax)	Not stated

*Mechanical complications were defined in accordance with the modified National Cancer Institute Common Terminology Criteria for Adverse Events, version 4.0. CICC, centrally inserted central venous catheter; CRBSI, catheter related blood stream infection; DVT, deep vein thrombosis; PICC, peripherally inserted central venous catheter; RRT, renal replacement therapy; TPN, total parenteral nutrition.

### Risk of clinically important infectious complication

Five trials (3,653 patients) were included in the analysis of clinically important infectious complications (3, 31, 32, 36, 37). Of these, four trials reported bloodstream infections (3, 31, 36, 37) and one trial reported catheter-related sepsis as a major catheter-related infectious complication (Supplementary Table 3) (32). Pairwise comparisons are presented in Supplementary Figure 1. The risk of bias was determined to be a concern for the outcome of infectious complications in one trial (Supplementary Table 4) (36). We did not rate down for the risk of bias, inconsistency, or publication bias (funnel plot shown in Supplementary Figure 2), but did for intransitivity (Supplementary Table 5). Incoherence between the direct and indirect RRs was not observed in any comparison.

**FIGURE 3:**
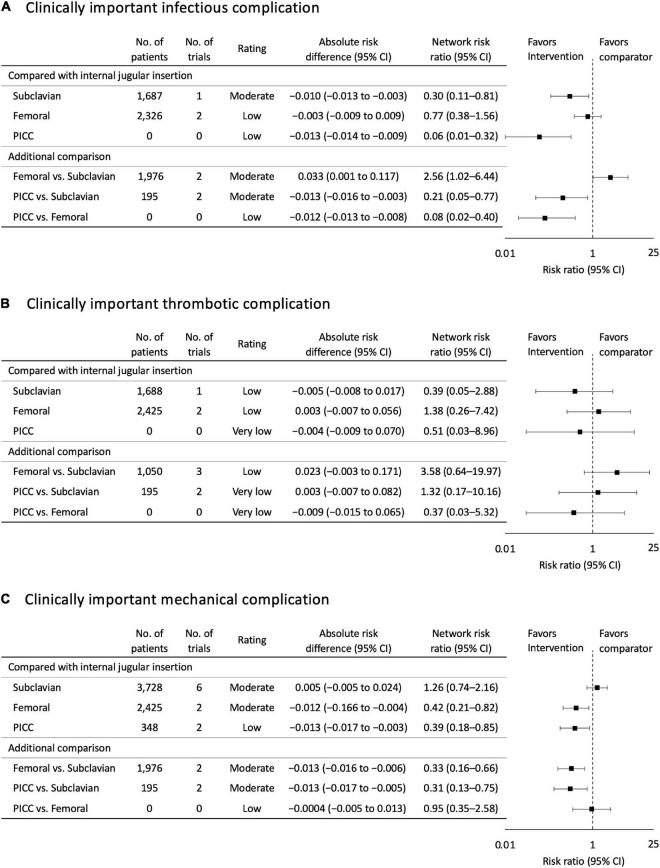
**(A)** Clinically important infectious complication. **(B)** Clinically important thrombotic complication. **(C)** Clinically important mechanical complication. Forest plots for association of central venous access sites with study outcomes. All outcomes are reported as network risk ratios and absolute risk differences with 95% confidence intervals (CIs). For estimating risk ratios for the comparison of peripherally inserted central venous catheter (PICC) vs. Internal jugular and PICC vs. Femoral, only indirect evidence was used, when no direct pair-wise comparisons were available. The estimated absolute risk was calculated based on the incidence of each outcome in patient allocated to the control group. CI, confidence interval; PICC, peripherally inserted central venous catheter.

Using internal jugular insertion as the reference, subclavian insertion [RR, 0.30 (95% CI, 0.11–0.81); risk difference (RD), −0.010 (95% CI, −0.013 to −0.003); moderate certainty] and PICC [RR, 0.06 (95% CI, 0.01-0.32); RD, −0.013 (95% CI, −0.014 to −0.009); low certainty] were significantly associated with a lower risk of infectious complications (Figure 3), but femoral insertion did not show a significant difference [RR, 0.06 (95% CI, 0.01–0.32); RD, −0.003 (95% CI, −0.009 to 0.009); low certainty].

**FIGURE 4:**
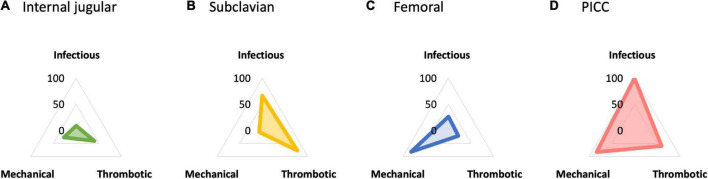
**(A)** Internal jugular insertion. **(B)** Subclavian insertion. **(C)** Femoral insertion. **(D)** Peripherally inserted central venous catheter (PICC). Radar chart plot of surface under the cumulative ranking curve (SUCRA) value of each complication among central venous access sites. The SUCRA value in reducing infectious complication was higher for peripherally inserted central venous catheter (PICC) (99.5), followed by subclavian (65.9), femoral (26.2), and internal jugular insertion (8.5). For thrombotic complication, subclavian insertion showed higher SUCRA value (77.8), followed by PICC (59.8), internal jugular (40.6), and femoral insertion (21.8). For mechanical complication, PICC showed higher SUCRA value (83.8), followed by femoral (82.2), internal jugular (27.3), and subclavian insertion (6.6). PICC, peripherally inserted central venous catheter; SUCRA, surface under the cumulative ranking curve.

For the additional comparison, femoral insertion increased the risk of infectious complications compared with subclavian insertion [RR, 2.56 (95% CI, 1.02–6.44); RD, 0.033 (95% CI, 0.001 to 0.117); moderate certainty]. PICC demonstrated a significant reduction in infectious complication risk compared with CICCs *via* other sites [compared with subclavian insertion: RR, 0.21 (95% CI, 0.05–0.77); RD, −0.013 (95% CI, −0.016 to −0.003); moderate certainty; compared with femoral insertion: RR, 0.08 (95% CI, 0.02–0.40); RD, −0.012 (95% CI, −0.013 to −0.008); low certainty]. The probability of being the best in reducing infectious complications among all possible insertion sites was higher for PICC, followed by subclavian, femoral, and internal jugular insertions (Figure 4 and Supplementary Figure 3).

### Risk of clinically important thrombotic complication

Six trials (3,813 patients) were included in the analysis of clinically important thrombotic complications (3, 31–33, 36, 37). Pairwise comparisons are presented in Supplementary Figure 1. Most of the included trials reported symptomatic venous thrombosis (Table 1). The risk of bias was determined to be high for the outcome of thrombotic complications in one trial (Supplementary Table 4) (33); however, we judged that the risk of bias was not serious because dominant trials had a low risk of bias. We did not rate down due to publication bias (funnel plot shown in Supplementary Figure 2), but we rated it down due to inconsistency in the comparison between subclavian insertion and PICC (Supplementary Table 5). Incoherence between the direct and indirect RRs was not observed in any comparison.

Using internal jugular insertion as the reference, subclavian insertion [RR, 0.39 (95% CI, 0.05–2.88); RD, −0.005 (95% CI, −0.008 to 0.017); low certainty] and PICC [RR, 0.51 (95% CI, 0.03–8.96); RD, −0.004 (95% CI, −0.009 to 0.070); very low certainty] decreased, and femoral insertion increased the risk of thrombotic complications [RR, 1.38 (95% CI, 0.26–7.42); RD, 0.003 (95% CI, −0.007 to 0.056); low certainty], although none of the comparisons were significant (Figure 3). Furthermore, there were no significant differences between the additional comparisons. The probability of being the best in reducing thrombotic complications among all possible insertion sites was higher for subclavian insertion, followed by PICC, internal jugular, and femoral insertions (Figure 4 and Supplementary Figure 3).

### Risk of clinically important mechanical complication

Twelve trials (6,140 patients) were included in the analysis of clinically important mechanical complications (3, 26–32, 34–37). Pairwise comparisons are presented in Supplementary Figure 1. Mechanical complications varied among the trials and included pneumothorax, hemothorax, hematoma, and serious bleeding. The risk of bias was determined to be high for the outcome of mechanical complications in five trials (Supplementary Table 4) (26, 27, 29, 34, 35), and we judged that the risk of bias was serious when comparing internal jugular insertion and PICC. We did not rate down due to publication bias (funnel plot shown in Supplementary Figure 2), but it was rated down due to inconsistency in the comparison between internal jugular and subclavian insertion (Supplementary Table 5). Incoherence between the direct and indirect RRs was not observed in any comparison.

Using internal jugular insertion as the reference, femoral insertion [RR, 0.42 (95% CI, 0.21–0.82); RD, −0.012 (95% CI, −0.166 to −0.004); moderate certainty] and PICC [RR, 0.39 (95% CI, 0.18-0.85); RD, −0.013 (95% CI, −0.017 to −0.003); low certainty] were significantly associated with a lower risk of mechanical complications (Figure 3), but subclavian insertion did not show a significant difference [RR, 1.26 (95% CI, 0.74–2.16); RD, 0.005 (95% CI, −0.005 to 0.024); moderate certainty].

For the additional comparison, femoral insertion decreased the risk of mechanical complications compared with subclavian insertion [RR, 0.33 (95% CI, 0.16–0.66); RD, −0.013 (95% CI, −0.016 to −0.006); moderate certainty] (Figure 3). PICC also decreased the risk of mechanical complications compared with subclavian insertion [RR, 0.31 (95% CI, 0.13–0.75); RD, −0.013 (95% CI, −0.017 to −0.005); moderate certainty], but did not show a significant difference compared with femoral insertion [RR, 0.95 (95% CI, 0.35–2.58); RD, −0.004 (95% CI, −0.005 to 0.013); low certainty]. The probability of being the best in reducing mechanical complications among all possible insertion sites was higher for PICC, followed by femoral, internal jugular, and subclavian insertions (Figure 4 and Supplementary Figure 3).

### Results of additional analyses

Both the sensitivity analysis that excluded trials enrolling CICCs *via* multiple insertion sites, and that which investigated catheter indwelling for ≤ 14 days revealed that PICC use was not associated with a lower incidence of infectious complications than with CICCs (Supplementary Tables 6, 7). However, most of these findings resulted from indirect comparisons between PICC and CICC, since only one trial comparing PICC with CICC *via* subclavian insertion was included in the sensitivity analysis.

For the analyses of patients with critical illness and catheter indwelling for ≤ 7 days, we could not evaluate the effect of PICCs since there were no trials comparing PICCs with CICCs in this population. Subclavian insertion was associated with a lower risk of infectious complications, and femoral insertion was associated with a lower risk of mechanical complications, similar to the results of the main analysis in critically ill patients (Supplementary Table 6). In patients with indwelling CICCs for 7 days or less, subclavian insertion was associated with a lower risk of infectious complications compared with internal jugular insertion, but not femoral insertion (Supplementary Table 7). No other *post hoc* sensitivity analyses were performed because no significant incoherence was observed for any outcome.

## Discussion

### Summary of evidence

In the current NMA of trials among adults with central venous catheterization, PICC decreased the risk of clinically important infectious complications compared with any other insertion site of the CICC. In addition, subclavian insertion was associated with a reduction in infectious complications compared to internal jugular and femoral insertions. Conversely, there were no significant differences in thrombotic complications among possible insertion sites. For the analysis of mechanical complications, PICC and femoral insertion were associated with a lower risk of mechanical complications as compared with insertion at other sites in CICCs. The fact that few RCTs compared PICC with CICC contributed to the lower certainty of evidence due to serious imprecision.

### Association with previous studies

A previous systematic review and meta-analysis demonstrated that subclavian and internal jugular insertion had similar risks for catheter-related complications in long-term catheterization (> 1 month) in patients with cancer, and femoral insertion increased catheter colonization and thrombotic complications, as compared to subclavian insertion (5). Thus, the recent clinical practice guidelines suggest the selection of an upper body insertion site to minimize the risk of infection in adult patients (38). In contrast, as per the results from the NMA of trials among critically ill patients conducted by Arvaniti et al. (7), colonization risk was higher for internal jugular [RR, 2.25 (95% CI, 1.84–2.75)] and femoral [RR, 2.92 (95% CI, 2.11–4.04)] insertion than for subclavian insertion. Our findings imply that internal jugular insertion may not be the best method for decreasing the risk of infectious complications, similar to the results of previous studies (3, 6, 39, 40).

Regarding thrombotic complications, femoral insertion was associated with a higher complication risk than subclavian insertion, but not internal jugular insertion as per the previous meta-analysis, although only one trial was included for each comparison (5). In an RCT with a large sample size comparing internal jugular, subclavian, and femoral insertion for CICCs, the thrombotic complication risk was higher for femoral insertion than for the other sites (3). We performed NMA using six RCTs, including these trials. Although there were no significant differences among the insertion sites, the confidence interval was wide, and the certainty of evidence was low in most comparisons. It is necessary to evaluate surrogate outcomes in current practice, because of the limited evidence for symptomatic thrombotic complications. Further RCTs are required to provide more conclusive evidence.

Mechanical complications are influenced by anatomical structures and are an important factor in the selection of insertion sites, even after short-term placement. Subclavian insertion is generally considered to be at the highest risk. According to the results of a previous meta-analysis, during short-term hemodialysis catheterization (< 1 month), internal jugular insertion was associated with a high risk of mechanical complications (5). Meanwhile, an interaction term between ultrasound guidance and insertion site has been reported (3). When performed without ultrasound guidance, femoral insertion decreased the risk of mechanical complications, but there were no significant differences among insertion sites for CICCs with ultrasound guidance. Our findings imply that femoral insertion is the best method to decrease the risk of mechanical complications. However, in more than half of the trials included in our analyses, the landmark technique was used for CICC insertion. Considering the current practice that ultrasound guidance is recommended, especially for internal jugular insertion (38), our results should be interpreted with caution in clinical application. Femoral insertion may not be the first choice but a preferable site for emergency cases, without ultrasound guidance, to avoid mechanical complications.

Peripherally inserted central venous catheters is expected to improve patient safety and has been widely used as an alternative to CICC in hospitalized patients (4). A meta-analysis comparing PICC with CICC demonstrated that PICC was associated with a lower risk of bloodstream infection (41); however, most trials were observational studies and only one RCT was included in this meta-analysis. Our NMA included four RCTs that evaluated PICC among patients receiving parenteral nutrition and chemotherapy. Our findings imply that PICC may be the best approach for decreasing the risk of major complications. However, most PICCs used in these trials were single or double lumens. Thus, the safety of PICCs in critically ill patients, who commonly require multi-lumen catheters, remains unclear, since multi-lumen and larger diameters may increase complication risks (42).

### Study implications and limitations

To our knowledge, no systematic reviews and meta-analyses have compared PICCs and CICCs according to their insertion sites. Furthermore, we present the risk of clinically important adverse events, including infectious, thrombotic, and mechanical complications. It may be difficult to judge based on only one of the complication risks, since the risk of complications and their value may vary among individuals (43). Our findings provide a rationale for deciding the insertion site for CICC.

However, the current NMA method has some limitations. First, in our NMA, most of the included trials enrolled critically ill patients, while some trials evaluating PICC did not include those patients, but patients with cancer. However, patients who receive chemotherapy are also at high risk of infectious and thrombotic complications, as are critically ill patients. In addition, critical illness may worsen mechanical complications, but may be less influential on occurrence. Second, catheter indwelling duration varied across the trials, especially in those comparing PICCs with CICCs. A *post hoc* analysis of catheter indwelling duration ≤ 14 days did not show a decrease in complication risk; however, only one comparative trial with PICCs was included in this analysis. Further study is needed to confirm the benefit of PICCs, even for a short duration. Third, the insertion technique and management methods, including antiseptics and ultrasound guidance or landmarks, varied. However, previous meta-analyses did not show a significant advantage of ultrasound guidance (44). In addition, chlorhexidine, which is effective in preventing infectious complications, was used in at least two RCTs, and povidone-iodine was used in several trials. Although managing insertion sites, including antiseptics, is also important for reduction in infection risks, the difference in antiseptics may have had little influence on insertion site comparisons, as the interaction between antiseptics and insertion sites is considered to be small (3). Considering these issues, the certainty of evidence for infectious complication was rated down because of intransitivity. Fourth, we need to note other unmeasured effect modifiers. For instance, most trials did not report the use of anticoagulants or antimicrobial agents, which may affect clinical outcomes. Fifth, there was a concern about the primary trials included in our review, regarding the lack of blinding of the interventional groups. Although this was unlikely to bias the assessment of hard outcomes based on the standardized definition, the assessment of soft outcomes and performance bias may be an important issue. Finally, the ranking results should be evaluated with caution because they do not consider the certainty of the evidence. Although PICC seemed to be the best choice when considering ranking probabilities, this result did not imply a significant clinical difference among the possible insertion sites. Further evaluation is needed, since few trials evaluating PICC with a sufficient sample size were found.

## Conclusion

The insertion site of the central venous catheter, which is most likely to cause the fewest complications, should be selected. Our findings can provide the rationale for deciding the insertion site for a central venous catheter, combined with baseline risk including patient risk, operator experience, and the expected duration of catheter placement. The current NMA demonstrated that PICC may be the most effective approach to avoid clinically important complication risks in hospitalized patients, despite with lower certainty. Considering the lower certainty on the safety of PICC, further studies are required to clarify whether PICC are preferable for hospitalized patients.

## Data availability statement

The raw data supporting the conclusions of this article will be made available by the authors, without undue reservation.

## Author contributions

MS designed the study, acquired data, performed the statistical analyses, and interpreted the data. HO and SY conceived the study and acquired and interpreted the data. SN and KK conceived the acquisition of data. The first draft of the manuscript was written by MS. All authors commented on previous versions of the manuscript, read, and approved the final manuscript.

## Footnotes

1
https://robotsearch.vortext.systems/#

## Supplementary material

Additional File 1 PRISMA network comparison meta-analysis (NMA) checklist.

Additional File 2 Supplementary information on the further results.
