# Supplementary material for: Insertion site of central venous catheter among hospitalized adult patients: A systematic review and network meta-analysis
Source: Front Med (Lausanne). 2022 Aug 29;9:960135. doi: 10.3389/fmed.2022.960135 (PMC9464814; doi:10.3389/fmed.2022.960135)

Supplementary Material

Insertion site of central venous access among hospitalized adult patients: a systematic review and network meta-analysis

Supplementary Figures and Tables

<u>Supplementary Tables</u>

Supplementary Table 1 Search strategy
………...…………..2–7

Supplementary Table 2 Additional characteristic details of the studies included in the network meta-analysis
……….………….8–9

Supplementary Table 3 The definition of clinically important infectious complication in the included studies
………..……………10

Supplementary Table 4 Summary of risk of bias of the studies included in the network meta-analysis
………..…………11–12

Supplementary Table 5 Summary of network meta-analysis and GRADE assessment for the effects of central venous access insertion site
…………………. 13–15

Supplementary Table 6 Pre-planned sensitivity analysis for the effects of central venous access insertion site
…………………. 16–17

Supplementary Table 7 Post-hoc sensitivity analysis according to catheter indwelling days
……..……………… 18

Figures

Supplementary Figure 1 Summary of random effects meta-analyses for direct comparisons
…………………. 19–21

Supplementary Figure 2 Comparison adjusted funnel plots for the network meta-analysis
…………………. 22–23

Supplementary Figure 3 Results of ranking probability in the network meta-analysis
…………………. 24–25

Supplementary Table 1 Search strategy

a. PubMed search strategy (search date: March 9, 2022)

Number	Searched for
#1	Catheterization, Central Venous [MeSH] OR Catheterization, Peripheral [MeSH] OR “central ven*” [tiab] OR CVC [tiab] OR PICC [tiab] OR Renal Replacement Therapy [MeSH] OR “renal replacement therapy” [tiab] OR HD [tiab] OR CHD [tiab] OR RRT [tiab] OR CVVHD OR CVVHDF OR CVVHF OR CHDF [tiab] OR HDF [tiab] OR dialysis [tiab] OR hemodialysis [tiab] OR Renal Dialysis [MeSH] OR Hemofiltration [MeSH] OR Hemofiltration [tiab]
#2	Jugular Veins [MeSH] OR Femoral Vein [MeSH] OR Subclavian Vein [MeSH] OR “insertion site” [tiab] OR "internal jugular*" [tiab] OR subclavian* [tiab] OR femoral* [tiab] OR peripheral* [tiab]
#3	Critical Illness [MeSH] OR “critical care” [tiab] OR “critically ill” [tiab] OR “intensive care” [tiab] OR ICU [tiab] OR Hospitals [MeSH] OR Hospital Units [MeSH] OR hospital [tiab] OR “care unit” [tiab]
#4	("Randomized Controlled Trial"[pt] OR "Controlled Clinical Trial"[pt] OR "Clinical Trials as Topic"[mh] OR randomized[tiab] OR placebo[tiab] OR randomly[tiab] OR trial[tiab] OR groups[tiab]) NOT (Animals [mh] NOT Humans [mh])
#5	#1 AND #2 AND #3 AND #4

b. CENTRAL search strategy (search date: March 9, 2022)

Number	Searched for
#1	MeSH descriptor: [Catheterization, Central Venous] explode all trees
#2	MeSH descriptor: [Catheterization, Peripheral] explode all trees
#3	(central venous):ti,ab,kw
#4	(CVC):ti,ab,kw
#5	(PICC):ti,ab,kw
#6	MeSH descriptor: [Renal Replacement Therapy] explode all trees
#7	(renal replacement therapy):ti,ab,kw
#8	(HD):ti,ab,kw
#9	(CHD):ti,ab,kw
#10	(RRT):ti,ab,kw
#11	(CHDF):ti,ab,kw
#12	(HDF):ti,ab,kw
#13	(CVVHD):ti,ab,kw
#14	(CVVHDF):ti,ab,kw
#15	(CVVHF):ti,ab,kw
#16	(dialysis):ti,ab,kw
#17	(hemodialysis):ti,ab,kw
#18	MeSH descriptor: [Renal Dialysis] explode all trees
#19	MeSH descriptor: [Hemofiltration] explode all trees
#20	(Hemofiltration):ti,ab,kw
#21	#1 OR #2 OR #3 OR #4 OR #5 OR #6 OR #7 OR #8 OR #9 OR #10 OR #11 OR #12 OR #13 OR #14 OR #15 OR #16 OR #17 OR #18 OR #19 OR #20
#22	MeSH descriptor: [Jugular Veins] explode all trees
#23	MeSH descriptor: [Femoral Vein] explode all trees
#24	MeSH descriptor: [Subclavian Vein] explode all trees
#25	(insertion site):ti,ab,kw
#26	(internal jugular*):ti,ab,kw
#27	(subclavian*):ti,ab,kw
#28	(femoral*):ti,ab,kw
#29	(peripheral*):ti,ab,kw
#30	#22 OR #23 OR #24 OR #25 OR #26 OR #27 OR #28 OR #29
#31	MeSH descriptor: [Critical Illness] explode all trees
#32	(critical care):ti,ab,kw
#33	(critically ill):ti,ab,kw
#34	(intensive care):ti,ab,kw
#35	(ICU):ti,ab,kw

#36	MeSH descriptor: [Hospitals] explode all trees
#37	MeSH descriptor: [Hospital Units] explode all trees
#38	(hospital):ti,ab,kw
#39	#31 OR #32 OR #33 OR #34 OR #35 OR #36 OR #37 OR #38
#40	#21 AND #30 AND #39

c. Web of science (search date: March 9, 2022)

Number	Searched for
S1	“Central venous Catheter” OR “Central venous Catheterization” OR CVC OR PICC OR “renal replacement therapy” OR HD OR CHD OR CVVHD OR CVVHDF ORCVVHF OR RRT OR CHDF OR HDF OR dialysis OR hemodialysis OR Hemofiltration
S2	“insertion site " OR jugular* OR subclavian* OR femoral* OR peripheral*
S3	“critical Illness” OR “critical care” OR “critically ill” OR “intensive care” OR ICU OR Hospital OR “care unit” OR ward
S4	"randomized controlled trial" OR "controlled clinical trial" OR randomized OR placebo OR "Drug Therapy" OR randomly OR trial OR groups NOT (animals NOT humans)

d. Ichushi search strategy (search date: March 9, 2022)

Number	Searched for
#1	(中心静脈カテーテル/TH OR 中心静脈カテーテル/AL) AND (PT=会議録除く)
#2	(中心静脈カテーテル法/TH OR CVC/AL) AND (PT=会議録除く)
#3	(PICC/AL) AND (PT=会議録除く)
#4	(血液透析/TH OR 透析/AL OR HD/AL) AND (PT=会議録除く)
#5	(腎置換療法/TH OR 腎代替療法/AL OR RRT/AL) AND (PT=会議録除く)
#6	((@血液濾過透析/TH AND @持続性腎置換療法/TH) OR CHDF/AL OR CHD/AL OR CVVHD/AL OR CVVHDF/AL OR CVVHF/AL) AND (PT=会議録除く)
#7	#1 OR #2 OR #3 OR #4 OR #5 OR #6
#8	(内頸静脈/TH OR 内頸静脈/AL) AND (PT=会議録除く)
#9	(鎖骨下静脈/TH OR 鎖骨下静脈/AL) AND (PT=会議録除く)
#10	(大腿静脈/TH OR 大腿静脈/AL) AND (PT=会議録除く)
#11	#8 OR #9 OR #10
#12	(入院患者/TH or 入院患者/AL) AND (PT=会議録除く)
#13	(ICU/TH or ICU/AL) AND (PT=会議録除く)
#14	(重症患者/AL) AND (PT=会議録除く)
#15	#12 OR #13 OR #14
#16	ランダム化比較試験/TH OR 準ランダム化比較試験/TH OR ランダム化/AL OR 無作為化/AL OR 比較試験/AL OR 臨床試験/AL OR プラセボ/AL OR 対照/AL OR コントロール/AL OR 臨床研究/AL
#17	#7 AND #11 AND #15 AND #16

e. Clinicaltrials.gov (search date: March 9, 2022)
Advanced search.
Condition or disease: “critical Illness” OR “critical care” OR “critically ill” OR “intensive care” OR ICU OR Hospital OR “care unit” OR ward OR “central venous catheter” OR “central venous catheterization” OR CVC OR PICC OR “renal replacement therapy” OR HD OR CHD OR RRT OR CHDF OR HDF OR CVVHD OR CVVHDF OR CVVHF OR dialysis OR hemodialysis OR Hemofiltration
Study type: interventional studies.
Study Results: Studied with Results
Intervention/treatment: (jugular OR subclavian OR femoral OR peripheral)
Age: adult (18–64 years) and Older Adult (65+)

f. WHO International Clinical Trials Registry Platform Search strategy (search date: March 9, 2022)
Advanced search
(jugular OR subclavian OR femoral OR peripheral) AND (“central venous catheter” OR “central venous catheterization” OR CVC OR PICC OR “renal replacement therapy” OR HD OR CHD OR RRT OR CHDF OR HDF OR CVVHD OR CVVHDF OR CVVHF OR dialysis OR hemodialysis OR Hemofiltration)

Supplementary Table 2 Additional characteristic details of the studies included in the network meta-analysis

Source	Age, y	Male, %	BMI, kg/m^2	Catheter placement days, d	Catheter type	Operator	Antiseptic	Dressing	Insertion technique	Antibiotic	Anticoagulant
Cowl 2000	59 *	55%	NA	CICC, 10.8; PICC, 9.6 *	CICC, up to triple lumens (5–7 F); PICC, single lumen (60 cm, 3 F) or double lumen (60 cm, 5.5 F)	Senior residents or specially trained nurses	Not stated	Sterile gauze and covered with a transparent membrane dressing	Not stated	Not stated	One patient with PICC received heparinization
Durbec 1997	41	77%	NA	8.5	CICC, single lumen, 60 cm, 2 mm	Not stated	Iodine solution	Covered with an occlusive dressing	Not stated	Not stated	Not stated
Fournil 2019	63	67%	26 *	NA	CICC	Not stated	Not stated	Not stated	US guidance	Not stated	Not stated
Gülmen 2009	60	73%	26.6	4.6	CICC	Same experienced physician	Povidone-iodine	Not stated	Landmark	Not stated	Not stated
Guo 2021	52	54%	NA	CICC, 27.3; PICC, 115.1	CICC, single-lumen (20 cm, 3.5 mm); PICC, single-lumen (60 cm, 5 F)	Nursing staff	Not stated	A sterile transparent dressing	Landmark	Not stated	Not stated
Kocum 2011	59	68%	27.7	NA	CICC, triple lumens (7 F, 20 cm)	Same experienced cardiovascular anesthesiologist	Chlorhexidine gluconate	Not stated	Landmark	Not stated	Not stated
Laiq 2015	42	68%	NA	NA	CICC	Not stated	Not stated	Not stated	Landmark	Not stated	Not stated
Merrer 2001	61	66%	23.7	10.1	CICC, up to triple lumens, 15–16cm	Physician (Senior physician 56%)	Local practice (Povidone-iodine 85%)	Semipermeable transparent dressing	Not stated	62.6%	85.1%

Parienti 2008	65	67%	26.7	6.5	Central venous access for RRT One center used antiseptics-impregnated catheters.	Trained physicians with at least 50 successful catheter insertions	Alcohol-based povidone-iodine	Not stated	Landmark, but US guidance was used in 1.9% of the patients with jugular insertion	Not stated	Used when indicated
Parienti 2015	63	64%	25.9	6.3	CICC, local practice	Resident or staff physician with at least 50 insertion experience	Local practice	Local practice	Landmark or US guidance	55.1%	30.2%
Picardi 2019	54 *	51%	NA †	NA	CICC, double or triple lumen (heparin coated); PICC, up to triple lumen (open-ended, nonvalved pressure injectable catheter with a flexible tip)	CVC, 3 intensivists; PICC, 6 medical hematology staffs	Local practice	Local practice	Always performing pre-scan, and real-time US guidance when possible	All patients were administered levofloxacin and posaconazole.	Not stated
Shin 2019	64 *	51%	23 *	2 *	CICC, double lumen	6 anesthesiologists with enough experience (> 200 internal jugular insertions and > 50 subclavian insertions within 2 y)	Chlorhexidine	Not stated	US guidance	Not stated	Not stated
Zhong 2021	56	58%	NA	NA	CICC, NA; PICC, single lumen	Not stated	Not stated	A transparent dressing	Not stated	Not stated	Not stated

* The value was reported as median.

† The proportion of obese patients (BMI $\geq$ 25 kg/m^2) was 12.9%.

List of abbreviations BMI, body mass index; CICC, centrally inserted central venous catheter; NA, not available; PICC, peripherally inserted central venous catheter; US, ultrasound.

Supplementary Table 3 The definition of clinically important infectious complication in the included studies

Source	The definition of catheter related infection
Cowl 2000	Positive cultures from both the catheter and a peripheral site
Merrer 2001	Major catheter-related infectious complications (probable catheter-related clinical sepsis without bloodstream infection and catheter-related clinical sepsis with bloodstream infection)
Parienti 2008	Catheter-tip colonization plus at least one peripheral blood culture yielding the same species with the same antimicrobial susceptibility as the catheter tip within 48 hours of catheter removal, with no other apparent source of sepsis
Parienti 2015	Catheter-tip colonization with the same phenotypic microorganism isolated from a peripheral blood culture
Picardi 2019	Either of the following: differential time to positivity of > 2 hours in a pair of central and peripheral blood cultures; detection of the same pathogen in a blood culture and at the catheter tip.

Supplementary Table 4 Summary of risk of bias of the studies included in the network meta-analysis

a. Clinically important infectious complication

Source	Bias arising from the randomization process	Bias due to deviations from intended interventions	Bias due to missing outcome data	Bias in measurement of the outcome	Bias in selection of the reported result	Overall risk of bias
Cowl 2000	Low risk	Some concerns	Low risk	Some concerns	Low risk	Some concerns
Merrer 2001	Low risk	Low risk	Low risk	Low risk	Low risk	Low risk
Parienti 2008	Low risk	Low risk	Low risk	Low risk	Low risk	Low risk
Parienti 2015	Low risk	Low risk	Low risk	Low risk	Low risk	Low risk
Picardi 2019	Low risk	Low risk	Low risk	Low risk	Low risk	Low risk

b. Clinically important thrombotic complication

Source	Bias arising from the randomization process	Bias due to deviations from intended interventions	Bias due to missing outcome data	Bias in measurement of the outcome	Bias in selection of the reported result	Overall risk of bias
Cowl 2000	Low risk	Some concerns	Low risk	High risk	Low risk	Some concerns
Durbec 1997	Some concerns	Some concerns	Low risk	High risk	Low risk	High risk
Merrer 2001	Low risk	Low risk	Low risk	High risk	Low risk	Low risk
Parienti 2008	Low risk	Low risk	Low risk	High risk	Low risk	Low risk
Parienti 2015	Low risk	Low risk	Low risk	High risk	Low risk	Low risk
Picardi 2019	Low risk	Low risk	Low risk	High risk	Low risk	Low risk

c. Clinically important mechanical complication

Source	Bias arising from the randomization process	Bias due to deviations from intended interventions	Bias due to missing outcome data	Bias in measurement of the outcome	Bias in selection of the reported result	Overall risk of bias
Cowl 2000	Low risk	Some concerns	Low risk	High risk	Low risk	Some concerns
Fournil 2019	Some concerns	Some concerns	Some concerns	Some concerns	Some concerns	High risk
Gülmen 2009	Some concerns	Some concerns	Low risk	Some concerns	Low risk	High risk
Guo 2021	Some concerns	Some concerns	Low risk	High risk	Low risk	High risk
Kocum 2011	Low risk	Some concerns	Low risk	Some concerns	Low risk	Some concerns
Laiq 2015	Some concerns	Some concerns	Low risk	Low risk	Low risk	High risk
Merrer 2001	Low risk	Low risk	Low risk	High risk	Low risk	Low risk
Parienti 2008	Low risk	Low risk	Low risk	High risk	Low risk	Some concerns
Parienti 2015	Low risk	Low risk	Low risk	High risk	Low risk	Low risk
Picardi 2019	Low risk	Low risk	Low risk	High risk	Low risk	Low risk
Shin 2019	Low risk	Low risk	Low risk	Some concerns	Low risk	Low risk
Zhong 2021	Some concerns	Some concerns	Low risk	High risk	Low risk	High risk

Supplementary Table 5 Summary of network meta-analysis and GRADE assessment for the effects of central venous access insertion site

a. Clinically important infectious complication

Comparison	Direct estimate (95% CI)	Rating	Indirect estimate (95% CI)	Rating	node split analysis (*P* value)	Network estimate (95% CI)	Rating
Subclavian vs Internal jugular	0.33 (0.11–1.03)	⊕⊕⊕◯ Moderate a)	0.21 (0.03–1.64)	⊕⊕◯◯ Low a), c)	0.638	0.30 (0.11–0.81)	⊕⊕⊕◯ Moderate a)
Femoral vs Internal jugular	0.76 (0.37–1.56)	⊕⊕⊕◯ Moderate a)	1.15 (0.04–38.5)	⊕◯◯◯ Very low b), c)	0.819	0.77 (0.38–1.56)	⊕⊕◯◯ Low b)
PICC vs Internal jugular	NA	NA	0.06 (0.01–0.32)	⊕⊕◯◯ Low a), c)	NA	0.06 (0.01–0.32)	⊕⊕◯◯ Low a), c)
Femoral vs Subclavian	2.65 (1.04–6.76)	⊕⊕⊕◯ Moderate a)	0.69 (0.00–200.00)	⊕◯◯◯ Very low b), c)	0.648	2.56 (1.02–6.44)	⊕⊕⊕◯ Moderate a)
PICC vs Subclavian	0.21 (0.05–0.77)	⊕⊕⊕◯ Moderate a)	NA	NA	NA	0.21 (0.05–0.77)	⊕⊕⊕◯ Moderate a)
PICC vs Femoral	NA	NA	0.08 (0.02–0.40)	⊕⊕◯◯ Low a), c)	NA	0.08 (0.02–0.40)	⊕⊕◯◯ Low a), c)

Testing for global incoherence P= 0.887

a) Serious imprecision, b) Very serious imprecision, c) Serious intransitivity

List of abbreviations CI, confidence interval; NA, not applicable; RR, risk ratio; PICC, peripherally inserted central venous catheter.

b. Clinically important thrombotic complication

Comparison	Direct estimate (95% CI)	Rating	Indirect estimate (95% CI)	Rating	node split analysis (*P* value)	Network estimate (95% CI)	Rating
Subclavian vs Internal jugular	0.50 (0.05–4.81)	⊕⊕○○ Low a)	0.19 (0.00–8.55)	⊕⊕○○ Low a)	0.726	0.39 (0.05–2.88)	⊕⊕○○ Low a)
Femoral vs Internal jugular	1.29 (0.24–6.85)	⊕⊕○○ Low a)	4.93 (0.00–1000)	⊕○○○ Very low a), b)	0.768	1.38 (0.26–7.42)	⊕⊕○○ Low a)
PICC vs Internal jugular	NA	NA	0.51 (0.03–8.96)	⊕○○○ Very low a), b)	NA	0.51 (0.03–8.96)	⊕○○○ Very low a), b)
Femoral vs Subclavian	3.77 (0.68–20.83)	⊕⊕○○ Low a)	1.15 (0.00–1000)	⊕⊕○○ Low a)	0.813	3.58 (0.64–19.97)	⊕⊕○○ Low a)
PICC vs Subclavian	1.32 (0.17–10.16)	⊕○○○ Very low a), b)	NA	NA	NA	1.32 (0.17–10.16)	⊕○○○ Very low b), c)
PICC vs Femoral	NA	NA	0.37 (0.03–5.32)	⊕○○○ Very low a), b)	NA	0.37 (0.03–5.32)	⊕○○○ Very low b), c)

Testing for global incoherence P= 0.970

a) Very serious imprecision, b) Serious inconsistency

List of abbreviations CI, confidence interval; NA, not applicable; RR, risk ratio; PICC, peripherally inserted central venous catheter.

c. Clinically important mechanical complication

Comparison	Direct estimate (95% CI)	Rating	Indirect estimate (95% CI)	Rating	node split analysis (*P* value)	Network estimate (95% CI)	Rating
Subclavian vs Internal jugular	1.25 (0.69–2.26)	⊕⊕◯◯ Low [a), b)]	1.31 (0.35–4.85)	⊕⊕⊕◯ Moderate [a)]	0.928	1.26 (0.74–2.16)	⊕⊕⊕◯ Moderate [a)]
Femoral vs Internal jugular	0.40 (0.19–0.84)	⊕⊕⊕◯ Moderate [a)]	0.50 (0.08–3.26)	⊕⊕◯◯ Low [a), b)]	0.970	0.42 (0.21–0.82)	⊕⊕⊕◯ Moderate [a)]
PICC vs Internal jugular	0.46 (0.20–1.08)	⊕⊕◯◯ Low [a), c)]	0.19 (0.03–1.13)	⊕⊕◯◯ Low [a), b)]	0.373	0.39 (0.18–0.85)	⊕⊕◯◯ Low [a), c)]
Femoral vs Subclavian	0.36 (0.16–0.82)	⊕⊕⊕◯ Moderate [a)]	0.24 (0.06–0.98)	⊕⊕◯◯ Low [a), b)]	0.842	0.33 (0.16–0.66)	⊕⊕⊕◯ Moderate [a)]
PICC vs Subclavian	0.16 (0.03–0.88)	⊕⊕⊕◯ Moderate [a)]	0.40 (0.14–1.09)	⊕⊕◯◯ Low [a), b)]	0.373	0.31 (0.13–0.75)	⊕⊕⊕◯ Moderate [a)]
PICC vs Femoral	NA	NA	0.95 (0.35–2.58)	⊕⊕◯◯ Low [a), b)]	NA	0.95 (0.35–2.58)	⊕⊕◯◯ Low [a), b)]

Testing for global incoherence P= 0.890

a) Serious imprecision, b) Serious inconsistency, c) Serious risk of bias

List of abbreviations CI, confidence interval; NA, not applicable; RR, risk ratio; PICC, peripherally inserted central venous catheter.

Supplementary Table 6 Pre-planned sensitivity analysis for the effects of central venous access insertion site

a. Clinically important infectious complication

comparison	Excluding trials that enrolled CICCs via multiple sites			Critically ill patients		
	No. of studies	RR (95% CI)	Rating	No. of studies	RR (95% CI)	Rating
Subclavian vs Internal jugular	1	0.30 (0.11–0.81)	Moderate [a)]	1	0.30 (0.11–0.81)	Moderate [a)]
Femoral vs Internal jugular	2	0.77 (0.38–1.56)	Moderate [a)]	2	0.77 (0.38–1.56)	Moderate [a)]
PICC vs Internal jugular	0	0.10 (0.00–2.80)	Very low [b), c)]	0	NA	NA
Femoral vs Subclavian	2	2.56 (1.02–6.44)	Moderate [a)]	2	2.56 (1.02–6.44)	Moderate [a)]
PICC vs Subclavian	1	0.33 (0.01–8.00)	Low [b)]	0	NA	NA
PICC vs Femoral	0	0.13 (0.00–3.56)	Very low [b), c)]	0	NA	NA

Testing for global incoherence: Excluding trials that evaluated CICCs with multiple sites, NA (fixed effect model); Only studies among critically ill patients, NA (fixed effect model). a) Serious imprecision, b) Very serious imprecision, c) Serious intransitivity

b. Clinically important thrombotic complication

comparison	Excluding trials that enrolled CICCs via multiple sites			Critically ill patients		
	No. of studies	RR (95% CI)	Rating	No. of studies	RR (95% CI)	Rating
Subclavian vs Internal jugular	1	0.40 (0.13–1.25)	Low [a)]	1	0.43 (0.13–1.31)	Low [a)]
Femoral vs Internal jugular	2	1.46 (0.65–3.27)	Low [a)]	2	1.44 (0.64–3.21)	Low [a)]
PICC vs Internal jugular	0	3.23 (0.31–33.39)	Very low [a), b)]	0	NA	NA
Femoral vs Subclavian	2	3.62 (1.28–10.25)	Low [a)]	3	3.33 (1.22–9.09)	Low [a)]
PICC vs Subclavian	1	8.00 (1.04–61.67)	Very low [a), b)]	0	NA	NA
PICC vs Femoral	0	2.21 (0.22–21.88)	Very low [a), b)]	0	NA	NA

Testing for global incoherence: Excluding trials that evaluated CICCs with multiple sites, P=0.885; Only studies among critically ill patients, NA (fixed effect model). a) Very serious imprecision, b) Serious inconsistency

c. Clinically important mechanical complication

comparison	Excluding trials that enrolled CICCs via multiple sites			Critically ill patients		
	No. of studies	RR (95% CI)	Rating	No. of studies	RR (95% CI)	Rating
Subclavian vs Internal jugular	6	1.20 (0.69–2.08)	Moderate [a)]	5	1.14 (0.64–2.02)	Moderate [a)]
Femoral vs Internal jugular	2	0.41 (0.20–0.80)	Moderate [a)]	2	0.40 (0.20–0.79)	Moderate [a)]
PICC vs Internal jugular	2	0.44 (0.20–1.00)	Low [a), b)]	0	NA	NA
Femoral vs Subclavian	2	0.34 (0.17–0.68)	Moderate [a)]	2	0.35 (0.17–0.70)	Moderate [a)]
PICC vs Subclavian	1	0.37 (0.14–0.97)	Moderate [a)]	0	NA	NA
PICC vs Femoral	0	1.09 (0.38–3.13)	Low [a), b)]	0	NA	NA

Testing for global incoherence: Excluding trials that evaluated CICCs with multiple sites, P= 0.969; Only studies among critically ill patients, P=0.961.

a) Serious imprecision, b) Serious risk of bias

List of abbreviations CI, confidence interval; NA, not applicable; RR, risk ratio; PICC, peripherally inserted central venous catheter.

Supplementary Table 7 Post-hoc sensitivity analysis according to catheter indwelling duration

a. Clinically important infectious complication

comparison	Catheter indwelling duration ≤ 14 d			Catheter indwelling duration ≤ 7 d		
	No. of studies	RR (95% CI)	Rating	No. of studies	RR (95% CI)	Rating
Subclavian vs Internal jugular	1	0.30 (0.11–0.81)	Moderate [a)]	1	0.32 (0.11–0.97)	Moderate [a)]
Femoral vs Internal jugular	2	0.77 (0.38–1.56)	Moderate [a)]	2	0.76 (0.37–1.56)	Moderate [a)]
PICC vs Internal jugular	0	0.10 (0.00–2.80)	Very low [b), c)]	0	NA	NA
Femoral vs Subclavian	2	2.56 (1.02–6.44)	Moderate [a)]	1	2.37 (0.76–7.36)	Moderate [a)]
PICC vs Subclavian	1	0.33 (0.01–8.00)	Low [b)]	0	NA	NA
PICC vs Femoral	0	0.13 (0.00–3.56)	Very low [b), c)]	0	NA	NA

Testing for global incoherence: Catheter indwelling duration ≤ 14 d, NA (fixed effect model); Catheter indwelling duration ≤ 7 d, NA (fixed effect model).

a) Serious imprecision, b) Very serious imprecision, c) Serious intransitivity

b. Clinically important thrombotic complication

comparison	Catheter indwelling duration ≤ 14 d			Catheter indwelling duration ≤ 7 d		
	No. of studies	RR (95% CI)	Rating	No. of studies	RR (95% CI)	Rating
Subclavian vs Internal jugular	1	0.43 (0.14–1.31)	Low [a)]	1	0.48 (0.15–1.56)	Low [a)]
Femoral vs Internal jugular	2	1.44 (0.64–3.21)	Low [a)]	2	1.40 (0.62–3.14)	Low [a)]
PICC vs Internal jugular	0	3.46 (0.34–35.33)	Very low [a), b)]	0	NA	NA
Femoral vs Subclavian	2	3.33 (1.22–9.09)	Low [a)]	1	2.91 (0.95–8.91)	Low [a)]
PICC vs Subclavian	1	8.00 (1.04–61.67)	Very low [a), b)]	0	NA	NA
PICC vs Femoral	0	2.40 (0.25–23.42)	Very low [a), b)]	0	NA	NA

Testing for global incoherence: Catheter indwelling duration ≤ 14 d, 0.885; Catheter indwelling duration ≤ 7 d, NA (fixed effect model).

a) Very serious imprecision, b) Serious inconsistency

List of abbreviations CI, confidence interval; NA, not applicable; RR, risk ratio; PICC, peripherally inserted central venous catheter.

Supplementary Figure 1 Summary of random effects meta-analyses for direct comparisons

Subclavian vs Internal jugular

a. Clinically important infectious complication

Study or Subgroup	Subclavian Events	Subclavian Total	Internal jugular Events	Internal jugular Total	Weight	Risk Ratio M-H, Random, 95% CI
Parienti 2015	4	843	12	845	100.0%	0.33 [0.11, 1.03]
**Total (95% CI)**		**843**		**845**	**100.0%**	**0.33 [0.11, 1.03]**
Total events	4		12			

Heterogeneity: Not applicable
Test for overall effect: Z = 1.91 (P = 0.06)

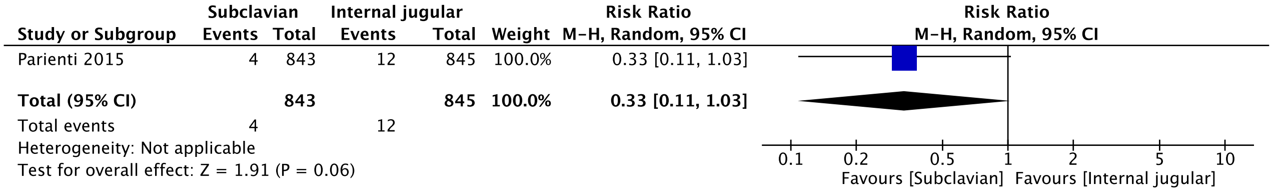

b. Clinically important thrombotic complication

Study or Subgroup	Subclavian Events	Subclavian Total	Internal jugular Events	Internal jugular Total	Weight	Risk Ratio M-H, Random, 95% CI
Parienti 2015	4	843	8	845	100.0%	0.50 [0.15, 1.66]
**Total (95% CI)**		**843**		**845**	**100.0%**	**0.50 [0.15, 1.66]**
Total events	4		8			

Heterogeneity: Not applicable
Test for overall effect: Z = 1.13 (P = 0.26)

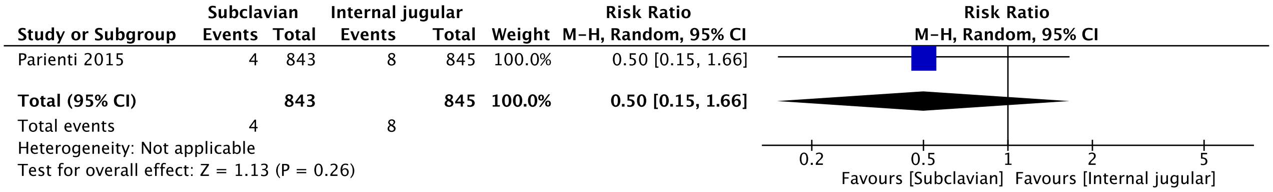

c. Clinically important mechanical complication

Study or Subgroup	Subclavian Events	Subclavian Total	Internal jugular Events	Internal jugular Total	Weight	Risk Ratio M-H, Random, 95% CI
Fournil 2019	3	101	2	100	18.2%	1.49 [0.25, 8.70]
Gülmen 2009	1	45	8	49	15.1%	0.14 [0.02, 1.05]
Kocum 2011	0	130	1	65	7.6%	0.17 [0.01, 4.07]
Laiq 2015	4	100	1	100	13.8%	4.00 [0.46, 35.16]
Parienti 2015	18	843	12	845	37.8%	1.50 [0.73, 3.10]
Shin 2019	1	677	0	673	7.5%	2.98 [0.12, 73.08]
**Total (95% CI)**		**1896**		**1832**	**100.0%**	**1.07 [0.41, 2.77]**
Total events	27		24			

Heterogeneity: $Tau^2 = 0.49$; $Chi^2 = 7.90$, df = 5 (P = 0.16); $I^2 = 37\%$
Test for overall effect: Z = 0.13 (P = 0.90)

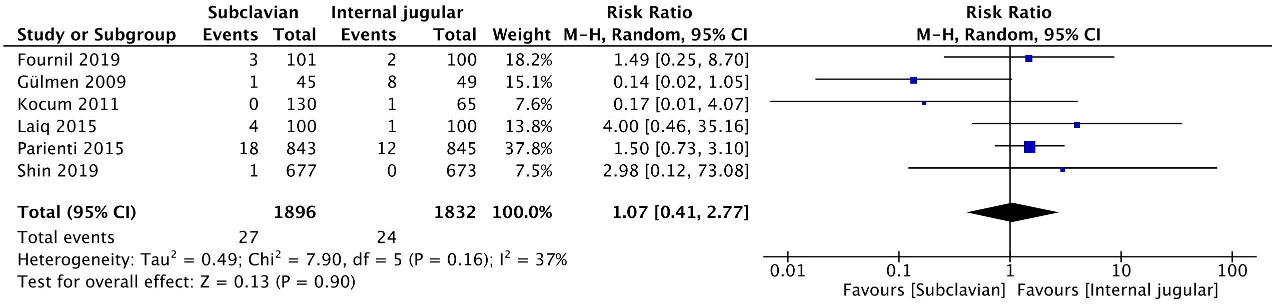

Femoral vs Internal jugular

a. Clinically important infectious complication

Study or Subgroup	Femoral Events	Femoral Total	Internal jugular Events	Internal jugular Total	Weight	Risk Ratio M-H, Random, 95% CI
Parienti 2008	3	324	5	313	25.6%	0.58 [0.14, 2.40]
Parienti 2015	10	844	12	845	74.4%	0.83 [0.36, 1.92]
**Total (95% CI)**		**1168**		**1158**	**100.0%**	**0.76 [0.37, 1.56]**
Total events	13		17			

Heterogeneity: $Tau^2 = 0.00$; $Chi^2 = 0.19$, df = 1 (P = 0.67); $I^2 = 0\%$
Test for overall effect: Z = 0.75 (P = 0.45)

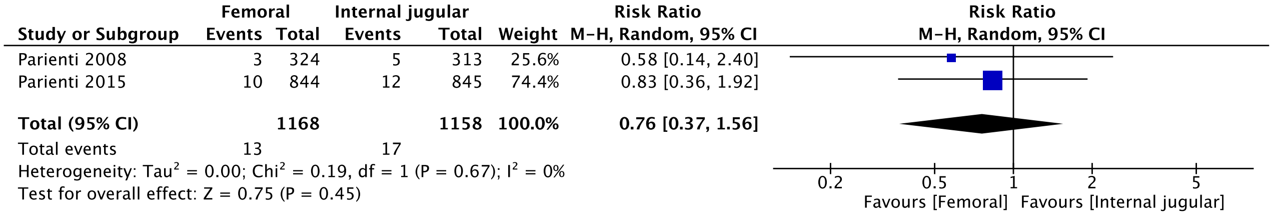

b. Clinically important thrombotic complication

Study or Subgroup	Femoral Events	Femoral Total	Internal jugular Events	Internal jugular Total	Weight	Risk Ratio M-H, Random, 95% CI
Parienti 2008	2	370	2	366	17.2%	0.99 [0.14, 6.98]
Parienti 2015	12	844	8	845	82.8%	1.50 [0.62, 3.66]
**Total (95% CI)**		**1214**		**1211**	**100.0%**	**1.40 [0.62, 3.14]**
Total events	14		10			

Heterogeneity: $Tau^2 = 0.00$; $Chi^2 = 0.15$, df = 1 (P = 0.70); $I^2 = 0\%$
Test for overall effect: Z = 0.81 (P = 0.42)

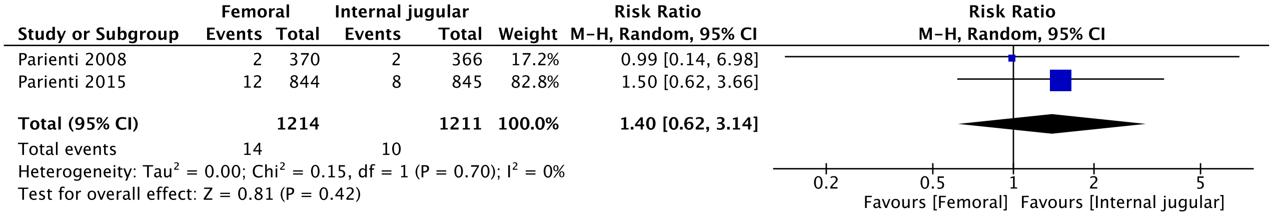

c. Clinically important mechanical complication

Study or Subgroup	Femoral Events	Femoral Total	Internal jugular Events	Internal jugular Total	Weight	Risk Ratio M-H, Random, 95% CI
Parienti 2008	4	370	13	366	43.5%	0.30 [0.10, 0.92]
Parienti 2015	6	844	12	845	56.5%	0.50 [0.19, 1.33]
**Total (95% CI)**		**1214**		**1211**	**100.0%**	**0.40 [0.19, 0.84]**
Total events	10		25			

Heterogeneity: Tau^2 = 0.00; Chi^2 = 0.44, df = 1 (P = 0.51); I^2 = 0%
Test for overall effect: Z = 2.43 (P = 0.02)

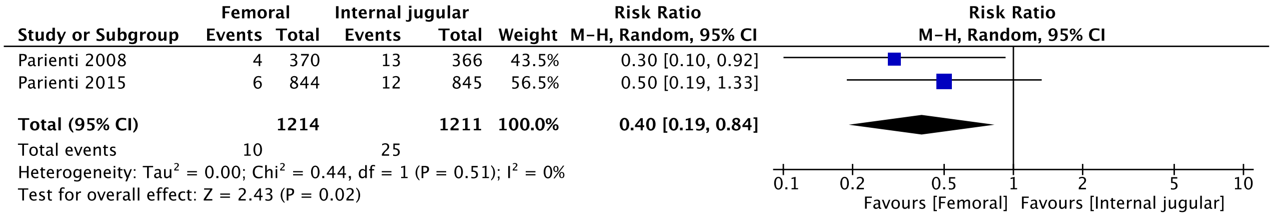

PICC vs Internal jugular

a. Clinically important infectious complication

Not applicable.

b. Clinically important thrombotic complication

Not applicable.

c. Clinically important mechanical complication

Study or Subgroup	PICC Events	PICC Total	Internal jugular Events	Internal jugular Total	Weight	Risk Ratio M-H, Random, 95% CI
Guo 2021	0	49	1	49	7.0%	0.33 [0.01, 7.99]
Zhong 2021	8	151	11	99	93.0%	0.48 [0.20, 1.14]
**Total (95% CI)**		**200**		**148**	**100.0%**	**0.46 [0.20, 1.08]**
Total events	8		12			

Heterogeneity: Tau^2 = 0.00; Chi^2 = 0.05, df = 1 (P = 0.83); I^2 = 0%
Test for overall effect: Z = 1.78 (P = 0.08)

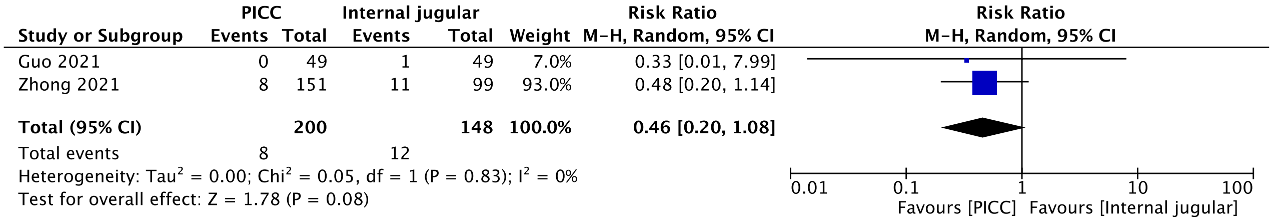

Femoral vs Subclavian

a. Clinically important infectious complication

Study or Subgroup	Femoral Events	Femoral Total	Subclavian Events	Subclavian Total	Weight	Risk Ratio M-H, Random, 95% CI
Merrer 2001	6	145	2	144	34.7%	2.98 [0.61, 14.52]
Parienti 2015	10	844	4	843	65.3%	2.50 [0.79, 7.93]
**Total (95% CI)**		**989**		**987**	**100.0%**	**2.66 [1.04, 6.75]**
Total events	16		6			

Heterogeneity: Tau^2 = 0.00; Chi^2 = 0.03, df = 1 (P = 0.86); I^2 = 0%
Test for overall effect: Z = 2.05 (P = 0.04)

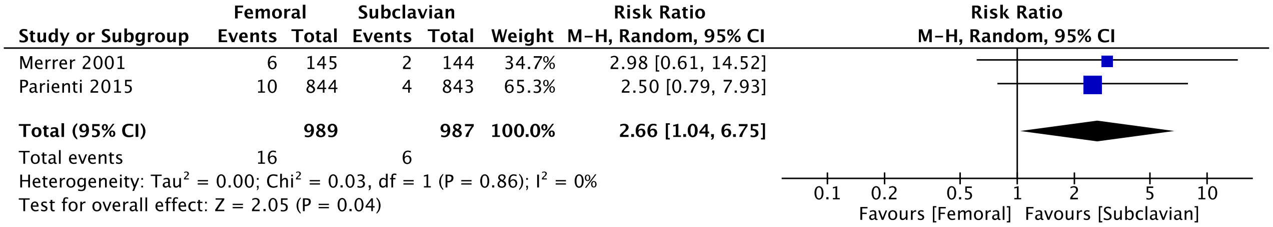

b. Clinically important thrombotic complication

Study or Subgroup	Femoral Events	Femoral Total	Subclavian Events	Subclavian Total	Weight	Risk Ratio M-H, Random, 95% CI
Durbec 1997	0	30	0	31		Not estimable
Merrer 2001	7	145	0	144	17.6%	14.90 [0.86, 258.44]
Parienti 2015	12	844	4	843	82.4%	3.00 [0.97, 9.25]
**Total (95% CI)**		**1019**		**1018**	**100.0%**	**3.98 [1.15, 13.77]**
Total events	19		4			

Heterogeneity: Tau^2 = 0.16; Chi^2 = 1.13, df = 1 (P = 0.29); I^2 = 11%
Test for overall effect: Z = 2.18 (P = 0.03)

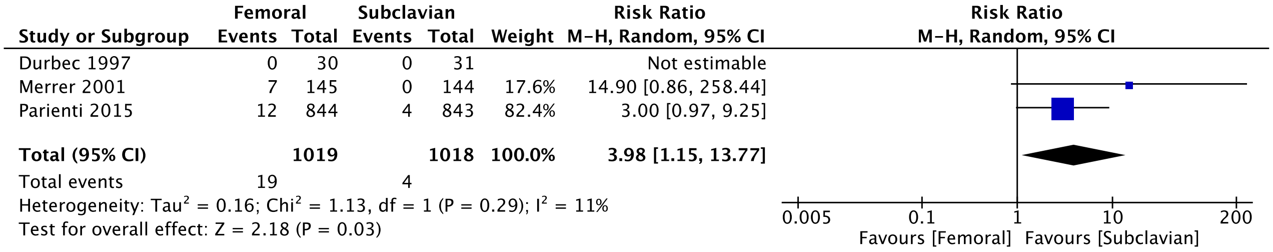

c. Clinically important mechanical complication

Study or Subgroup	Femoral Events	Femoral Total	Subclavian Events	Subclavian Total	Weight	Risk Ratio M-H, Random, 95% CI
Merrer 2001	2	145	4	144	23.0%	0.50 [0.09, 2.67]
Parienti 2015	6	844	18	843	77.0%	0.33 [0.13, 0.83]
**Total (95% CI)**		**989**		**987**	**100.0%**	**0.36 [0.16, 0.82]**
Total events	8		22			

Heterogeneity: Tau^2 = 0.00; Chi^2 = 0.17, df = 1 (P = 0.68); I^2 = 0%
Test for overall effect: Z = 2.45 (P = 0.01)

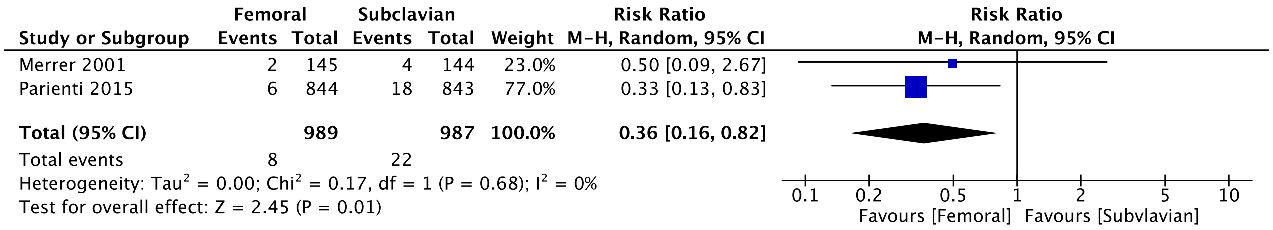

PICC vs Subclavian

a. Clinically important infectious complication

Study or Subgroup	PICC Events	PICC Total	Subclavian Events	Subclavian Total	Weight	Risk Ratio M-H, Random, 95% CI
Cowl 2000	0	51	1	51	17.3%	0.33 [0.01, 8.00]
Picardi 2019	2	46	11	47	82.7%	0.19 [0.04, 0.79]
**Total (95% CI)**		**97**		**98**	**100.0%**	**0.21 [0.05, 0.77]**
Total events	2		12			

Heterogeneity: Tau^2 = 0.00; Chi^2 = 0.11, df = 1 (P = 0.74); I^2 = 0%
Test for overall effect: Z = 2.35 (P = 0.02)

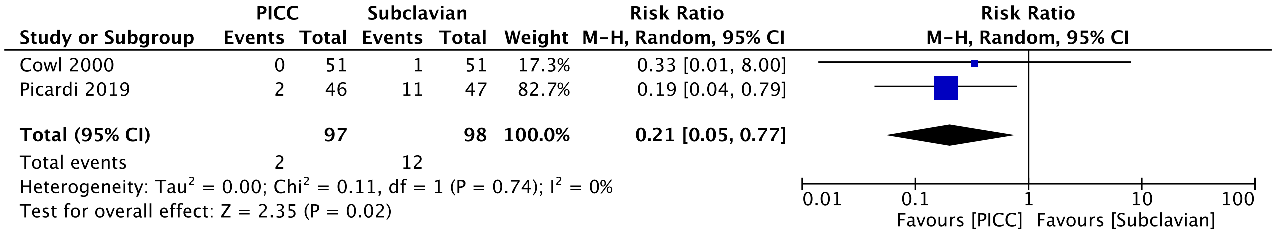

b. Clinically important thrombotic complication

Study or Subgroup	PICC Events	PICC Total	Subclavian Events	Subclavian Total	Weight	Risk Ratio M-H, Random, 95% CI
Cowl 2000	8	51	1	51	50.3%	8.00 [1.04, 61.67]
Picardi 2019	1	46	5	47	49.7%	0.20 [0.02, 1.68]
**Total (95% CI)**		**97**		**98**	**100.0%**	**1.29 [0.04, 47.28]**
Total events	9		6			

Heterogeneity: Tau^2 = 5.63; Chi^2 = 6.02, df = 1 (P = 0.01); I^2 = 83%
Test for overall effect: Z = 0.14 (P = 0.89)

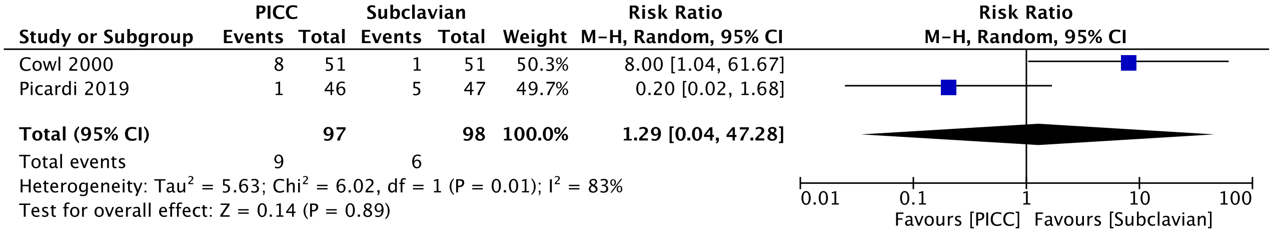

c. Clinically important mechanical complication

Study or Subgroup	PICC Events	PICC Total	Subclavian Events	Subclavian Total	Weight	Risk Ratio M-H, Random, 95% CI
Cowl 2000	0	51	2	51	31.8%	0.20 [0.01, 4.07]
Picardi 2019	1	46	7	47	68.2%	0.15 [0.02, 1.14]
**Total (95% CI)**		**97**		**98**	**100.0%**	**0.16 [0.03, 0.88]**
Total events	1		9			

Heterogeneity: Tau^2 = 0.00; Chi^2 = 0.03, df = 1 (P = 0.87); I^2 = 0%
Test for overall effect: Z = 2.11 (P = 0.04)

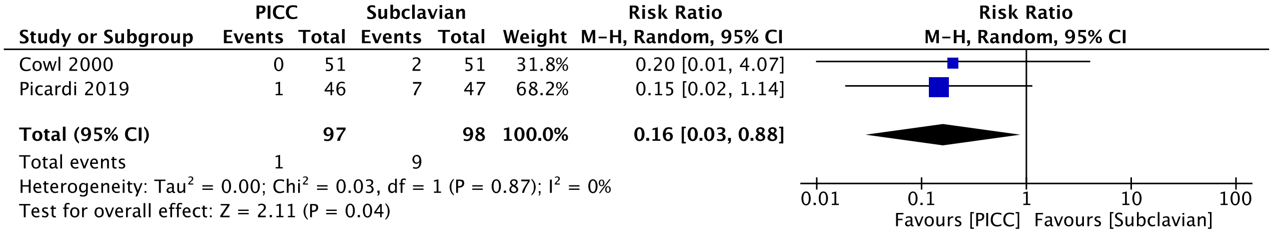

List of abbreviations CI, confidence interval; PICC, peripherally inserted central venous catheter.

Supplementary Figure 2 Comparison adjusted funnel plots for the network meta-analyses

a. Clinically important infectious complication

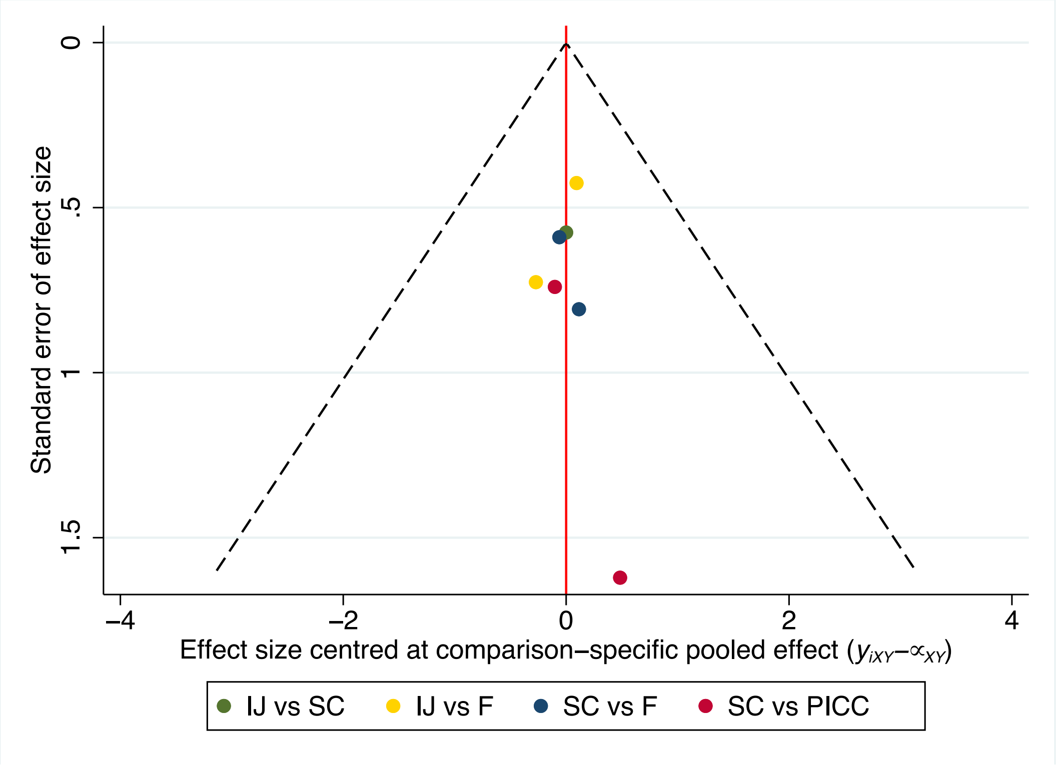

b. Clinically important thrombotic complication

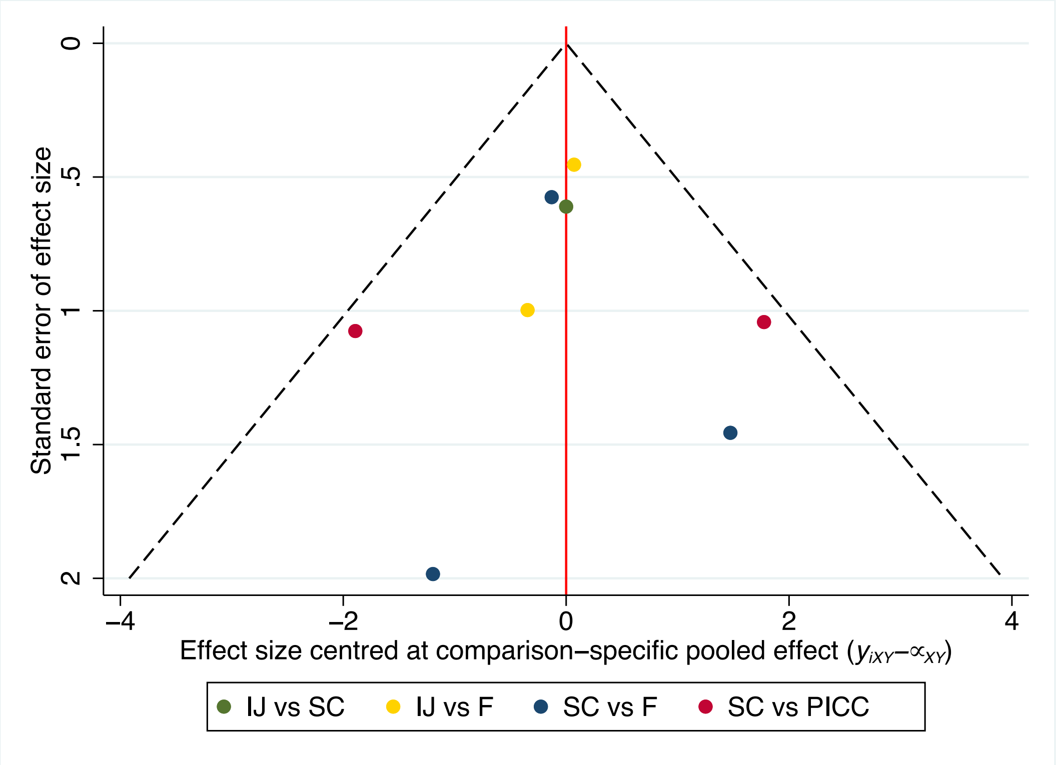

List of abbreviations F, femoral; IJ, internal jugular; PICC, peripherally inserted central venous catheter; SC, subclavian.

c. Clinically important mechanical complication

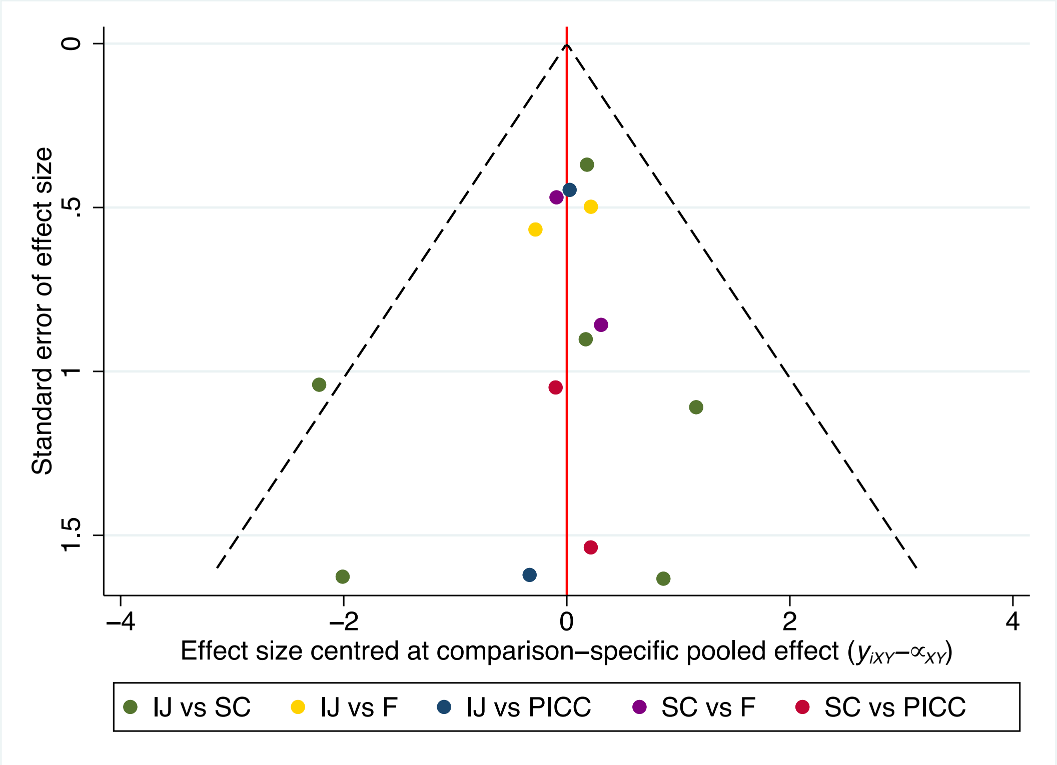

List of abbreviations F, femoral; IJ, internal jugular; PICC, peripherally inserted central venous catheter; SC, subclavian.

Supplementary Figure 3 Results of ranking probability in the network meta-analysis

a. Clinically important infectious complication

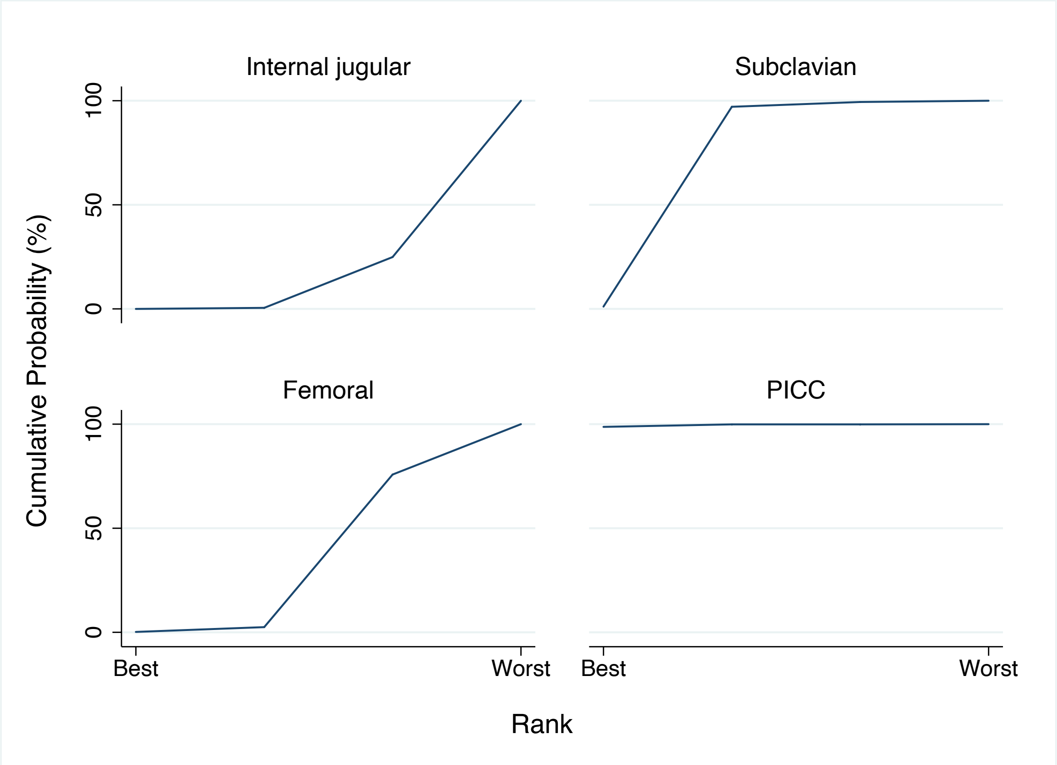

b. Clinically important thrombotic complication

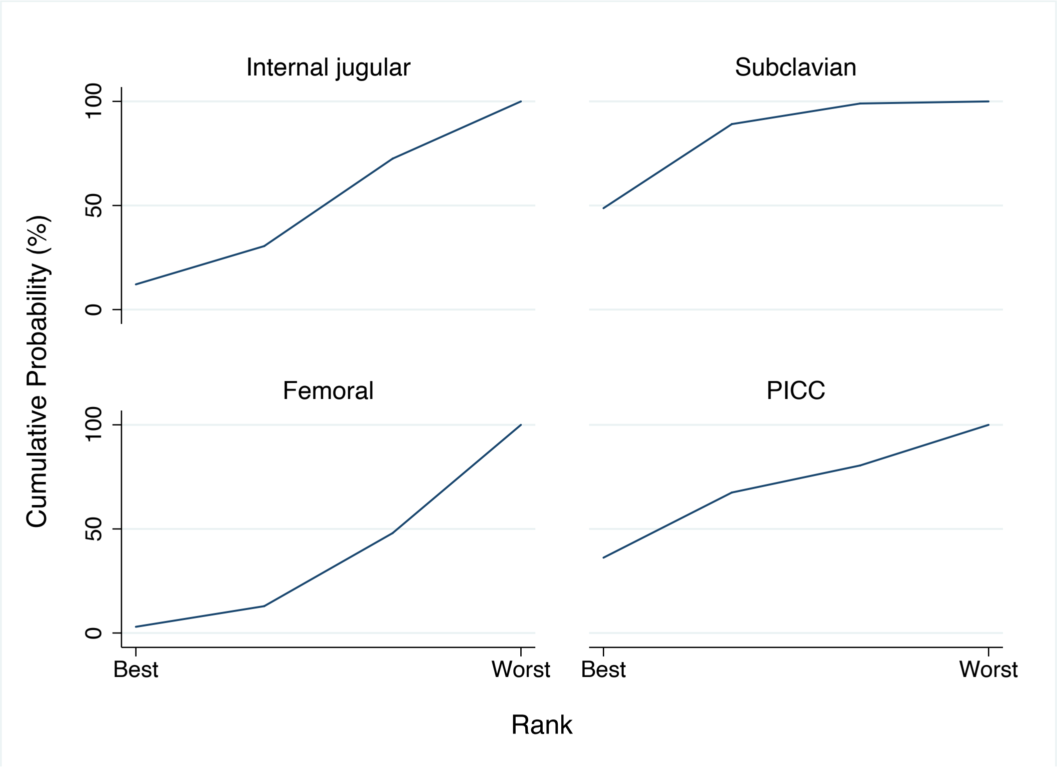

c. Clinically important mechanical complication

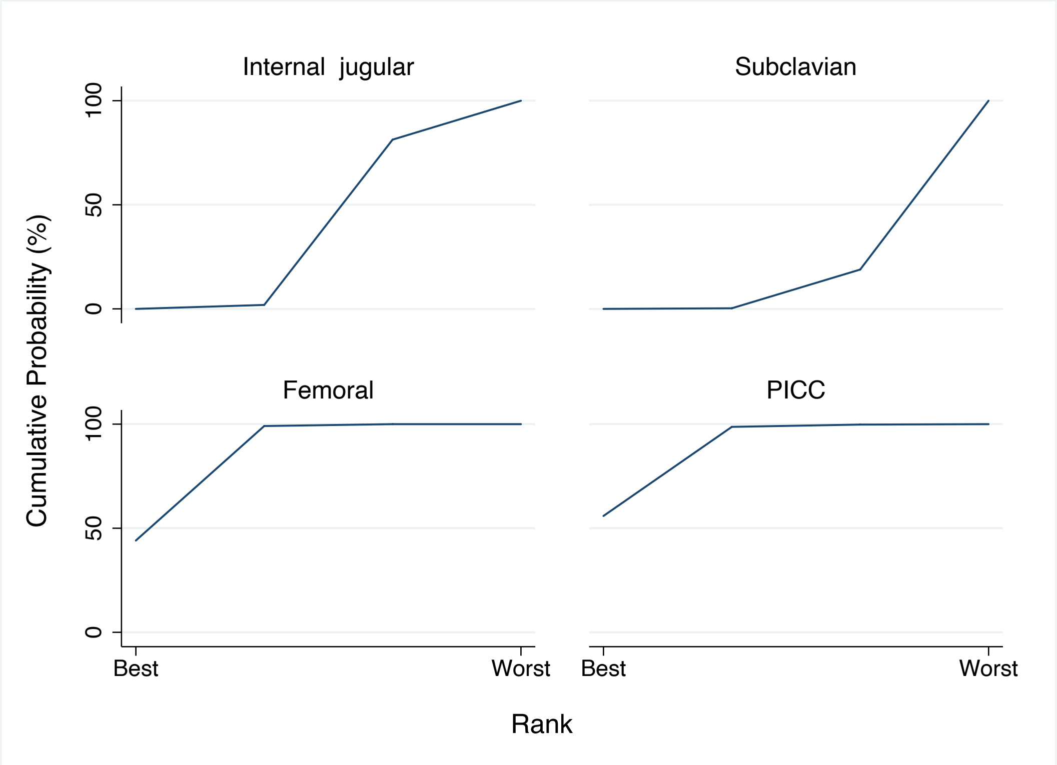

Supplement: Additional File 1 — PRISMA network comparison meta-analysis (NMA) checklist. [file Data_Sheet_1.PDF]
